# Longitudinal Active Avian Influenza Surveillance in Bangladesh From 2017–2022 Reveals Differential IAV and H5 Infection and Viral Burden Associated With Bird Species, Sex, and Age

**DOI:** 10.1155/tbed/5569836

**Published:** 2024-12-13

**Authors:** Walter N. Harrington, Jasmine C. M. Turner, Subrata Barman, Mohammed M. Feeroz, Md. Kamrul Hasan, Sharmin Akhtar, Trushar Jeevan, Nabanita Mukherjee, Patrick Seiler, John Franks, David Walker, Pamela McKenzie, Lisa Kercher, Robert G. Webster, Richard J. Webby

**Affiliations:** ^1^Department of Host-Microbe Interactions, St. Jude Children's Research Hospital, Memphis, Tennessee, USA; ^2^Department of Zoology, Jahangirnagar University, Savar, Bangladesh

**Keywords:** avian influenza, pandemic preparedness, risk assessment, sex-specific bias, surveillance

## Abstract

Influenza viruses are a major global health burden with up to 650,000 associated deaths annually. Beyond seasonal illness, influenza A viruses (IAVs) pose a constant pandemic threat due to novel emergent viruses that have evolved the ability to jump from their natural avian hosts to humans. Because of this threat, active surveillance of circulating IAV strains in wild and domestic bird populations is vital to our pandemic preparedness and response strategies. Here, we report on IAV surveillance data collected from 2017 to 2022 from wild and domestic birds in Bangladesh. We note evidence to suggest that male birds show a higher risk of IAV, including highly pathogenic avian influenza (HPAI) A(H5) virus, positivity than female birds. The data was stratified to control for selection bias and confounding variables to test the hypothesis that male birds are at a higher risk of IAV positivity relative to female birds. The association of IAV and A(H5) largely held in each stratum, and double stratification suggested that the phenomena was largely specific to ducks. Finally, we show that chickens, male birds, and juvenile birds generally have higher viral loads compared to their counterparts. These observations warrant further validation through active surveillance across various populations. Such efforts could significantly contribute to the enhancement of pandemic prediction and risk assessment models.

## 1. Introduction

The influenza virus is an important public health threat that is associated with 3–5 million severe cases and 290,000–650,000 deaths annually [1, 2]. There are two major genera of disease-causing influenza viruses in humans, influenza A virus (IAV) and influenza B virus (IBV) [3]. Where the only known reservoir of IBV is humans, the natural reservoir of IAV is aquatic birds [4–7]. IAV circulate and evolve in this natural reservoir and then make occasional jumps into humans, some jumps resulting in pandemics. There have been four influenza pandemics in the last 110 years, and the pandemic threat is ever present [8]. Although there are 18 known subtypes of IAV (H1-H18) that have been isolated from avian (H1–H16) and bat (H17–H18) species, only H1, H2, and H3 subtypes have been known to cause pandemics in humans [9–11]. However, in recent years human infections with H5, H7, and H9 viruses have increased concern that one of these subtypes might become the next influenza pandemic [12, 13].

IAV strains typically evolve antigenically via two mechanisms, antigenic drift and antigenic shift [3, 14–17]. Antigenic drift occurs as the IAV strain accumulates point mutations in key antigenic domains leading to escape from immune system pressures. Antigenic shift, or reassortment, occurs when two different strains of IAV coinfect the same host and reassort to form a novel strain, made possible by the segmented nature of the influenza virus genome. Antigenic shift drives IAV pandemic emergence, as the novel strains have little to no population immunity in humans [17, 18]. Various IAV cocirculate within wild and domestic bird populations, producing ample opportunity for reassortment to occur. Therefore, active influenza virus surveillance within different bird populations is a vital aspect of pandemic preparedness and rapid pandemic response [6, 18–20]. Furthermore, understanding how various host bird characteristics impact influenza susceptibility, such as sex and age, will help inform pandemic prediction models and target risk assessment processes.

The country of Bangladesh is located in Asia and is situated in two major overlapping migratory bird flyways, the Central Asian flyway and the East Asian–Australian flyway [21–23]. The country also has many poultry farms, both backyard and commercial, which enables interaction and transmission of IAV strains between wild and domestic birds [21]. Backyard and free-range duck farms in particular facilitate contact between wild and domestic birds [24]. Further, it has been shown that poor biosecurity and hygiene practices in live bird markets (LBMs) in Bangladesh drive IAV transmission and novel strain emergence [25–27]. Bangladesh also has one of the highest human density populations in the world, increasing the numbers of individuals exposed to infected birds and allowing rapid transmission should a strain gain the ability to infect humans [25, 26, 28]. Bangladesh is, therefore, an ideal location to target for longitudinal studies of wild and domestic birds in various regions and habitats to better understand which strains are currently circulating and what factors drive zoonotic and pandemic risk in these complex environemnts [26, 29].

Here, we analyze a dataset gathered from samples taken in Bangladesh from 2017 to 2022 to identify influences of sex, age, and species in viral prevalence and viral RNA load.

## 2. Materials and Methods

### 2.1. Collection Methods

Swabs from live birds (cloacal, oral–pharyngeal, and combined cloacal and oral–pharyngeal) and the environment (feces and cage water) were taken from birds from backyard and commercial farms, in Bangladesh from late November 2016 through December 2022. A detailed method for these collections has been published previously [29, 30]. All samples and metadata were collected and recorded in Bangladesh and then sent to St. Jude for further analysis. Real-time reverse transcriptase polymerase chain reaction (qRT-PCR) assay using the CDC defined primers and probe for IAV matrix (M) gene [31] (FWD 1:5′-CAA GAC CAA TCY TGT CAC CTC TGA C-3′, FWD 2:5′-CAA GAC CAA TYC TGT CAC CTY TGA C-3′, REV 1:5′-GCA TTY TGG ACA AAV CGT CTA CG-3′, REV 2:5′-GCA TTT TGG ATA AAG CGT CTA CG-3′, PROBE InfA-P: 5′-TGC AGT CCT CGC TCA CTG GGC ACG-3′) to determine the influenza A status of the sample before attempt at egg isolation. Samples positive for IAV were then probed with H5 HA (kit available through CDC International Reagent Resource (IRR) [32]) gene primers and probes to determine if they were positive for highly pathogenic avian influenza (HPAI) H5Nx. Cycle threshold (Ct) values obtained through qRT-PCR have been used as a metric for viral burden. Field data obtained were used for further statistical analyses with Python utilizing the Pandas framework (Anaconda, Inc.). All samples were processed in accordance with St. Jude IBC protocols 02 A-221 and 03-222.

### 2.2. Data Cleaning

The initial dataset consisted of a total of 24,598 avian samples. Three hundred thirty of these samples were not screened for influenza and were removed from the analyses, leaving 24,268 samples. For 8398 of these samples, two swabs (one cloacal and one oropharyngeal) were taken from the same bird, resulting in sampling duplication. To reconcile and streamline data for analyses, deduplication measures were employed. In brief, if both CLO and ORP swabs were positive or negative, only the ORP swab was considered in the subsequent analysis. If only one of the CLO or ORP swabs was positive, only the positive swab was considered for subsequent analysis. The deduplication measures resulted in a final 20,069 distinct host samples to be analyzed. Wild birds were almost exclusively sampled of by environmental sampling (7728 fecal, 50 water, 18 combined oral and cloacal, and 474 sentinel bird sampling). Domestic chickens and quail were exclusively sampled by oropharyngeal swabs (2708 and 800, respectively), except for five quail that were sampled by cloacal swabs. Domestic ducks were primarily sampled by oropharyngeal swabs (5268), followed by cloacal swabs (1503), combined oral and cloacal swabs (450), and environmental sampling (246). Water samples were taken from cages of chickens (1350), ducks (60), and quail (195).

### 2.3. Descriptive Analysis and Data Stratification

Metadata was collected for each of the samples, including the collection date, farming classification, bird type (wild or domestic), habitat, location, age, sample type, sex, species, and health status. Python (Jupyter Notebook v.7.0.8 through Anaconda Navigator v2.6.2, using Pandas v2.0.0) was used to clean analyze the dataset [33, 34]. Code and data files have been made available on the Open Science Framework [35]. *χ*^2^ tests with multiple comparisons were used to test differences in IAV and H5 positivity within each metadata group using a python script [36]. The significance threshold was set at an alpha level of 0.05. A reference group was chosen within each metadata group to test differences using multiple comparisons. Logistic regression could not be performed on the data due to the nature of the sampling and interferences of variables. The data was instead stratified on relevant variables to decrease the effect of sampling bias, allowing variables of interest to be compared within relatively similar subgroups of birds. Two variables, sex and age classification, were selected for stratification based on their unexpected influence of IAV and H5 positivity. These variables were analyzed in groups keeping sample type, habitat, age (for age analysis), sex (for sex analysis), species, location, health status, and combinations thereof constant. Real-time qRT-PCR Ct values were also analyzed for several species in the dataset as a whole and in the LBM habitat strata, in particular. Significant differences between qRT-PCR Ct values were determined by pairwise *t*-tests at an alpha level of 0.05.

### 2.4. qRT-PCR and Ct Value Distribution Analysis

Real-time qRT-PCR targeting the M gene of IAV yielded Ct cutoff values. These values were then used to determine differences in viral load in various categories and subcategories of our data. For each category, a distribution of Ct values was created and plotted for visualization. Two sample Kolmogorov–Smirnov tests were used to determine significant differences between distributions at an alpha level of 0.05. Real-time qRT-PCR primers and probes specific to H5 were also used to determine H5 positivity.

## 3. Results

### 3.1. Descriptive Statistics

Seven metadata categories and two LBM subcategories were summarized and analyzed per subgroup for differences in IAV and H5 positivity: sample type, habitat, source of bird (LBM), age, sex, sex (LBM), host species, location, and health status. The groups were analyzed both as a whole and in comparison, with a noted reference group using *χ*^2^ tests with multiple comparisons.  Table 1 shows the findings of our initial analysis. At this high-level view, there were many significant differences within the categories analyzed, such that no variable was completely without differences within its own group. Unsurprisingly, cloacal swabs and retail birds (in LBMs) were associated with the highest IAV positivity (56.0% and 46.8%, respectively) within their metadata group. This finding largely held for H5 positivity within the habitat group, but there were fewer differences in H5 positivity within sample type. LBM samples that came from birds sourced from backyard farms had higher IAV (53.4% vs. 44.2%) and H5 (23.4% vs. 8.7%) positivity compared to birds sourced from commercial farms. Chicken and quail showed the highest IAV positivity rates (42.7% and 43.9%, respectively), followed closely by duck (37.5%). Environmental (fecal) samples had the lowest IAV and H5 positivity rates (12.4% and 0.0%, respectively). It is important to note that the vast majority (99.8%) of these environmental samples were collected from wild birds rather than farm or market birds, which may reflect the overall lower infection rates among the wild bird population sampled.

The market collection locations (Market 1, Market 2, Market 3, and Market 4) had elevated IAV positivity rates (38.1%−56.5%) compared to non-market location (Tanguar Hoar, Farm 1, Farm 2, Farm 3, and the Lake) rates (8.0%−30.7%). H5 positivity rates also varied throughout the markets, with Markets 3 and 4 having the highest rates (19.6% and 18.1%, respectively), and Farm 1 having the lowest rate (0.6%), which was comparable to the environmental samples from wild birds in Tanguar Haor (0.1%) and the Lake (0.7%).

Most of the samples were taken from apparently healthy birds, which had higher IAV positivity rates than sick birds (42.0% vs. 23.3%). Birds that were identified as sick (not specifically IAV) had lower positivity rates; dead birds had the same level of IAV positivity (42.3%) as healthy birds (*p* ≥ 0.05). Birds of undetermined health status were almost exclusively environmental (fecal) samples from wild birds.

The two most surprising findings in this data set were the significant differences between male and female birds for both IAV positivity (46.5% vs. 38.2%, respectively; *p*  < 0.0001) and H5 positivity (16.5% vs. 3.6%, respectively; *p*  < 0.0001), and the higher IAV positivity rates in birds classified as after hatch year (AHY) compared to hatch year (HY) (41.9% vs. 34.5%, respectively; *p*  < 0.0001). H5 positivity rates showed the opposite trend in these age classifications. The sex and age differences prompted us to determine if these differences were valid inferences from the data or if there was selection or confounding bias that exaggerated the association.

### 3.2. Bird Sex and IAV/H5 Positivity

In our initial analysis, we found that samples from male birds were significantly more likely to be RT-PCR positive than female birds. An even stronger association between sex and infection was suggested by the H5 data. This finding was surprising but could be explained by sampling bias due to our collection methods and locations (e.g., if the more females were sampled on farms where IAV prevalence is lower and more males were sampled in markets where IAV prevalence is higher). To limit this bias, we stratified the data on several variables (sample type, habitat, age, species, location, and health status) and looked specifically at the male/female differences in IAV and H5 positivity rates in each stratum (Table 2). All samples of unknown sex were removed from this analysis. It should be noted that virtually all (99.7%) wild duck samples were environmental samples from which sex could not be determined; therefore, sex stratification was limited to domestic poultry.

Following stratification, the significance of the difference between IAV and H5 positivity rates in male and female birds diminished in some instances. Nevertheless, notable differences persisted. These differences were less conspicuous in the “Sample type” category, where two out of four subcategories exhibited an inverse relationship: cloacal swabs (60.3% IAV positivity in females vs. 46.1% in males; *p*  > 0.001) and water samples (46.5% vs. 40.3%, respectively; *p*  < 0.05). However, in all other stratifications except for samples collected at Market 3, IAV positivity was either higher or not significantly different in male birds compared to female birds. Age classification did not appear to play a biasing factor, as the association remained across both age classifications. Notably, across all stratifications, H5 positivity rates were either higher or not significantly different in male birds.

Stratification by habitat and species provided insights into the association between sex and IAV positivity. Birds within the same Habitat stratum are likely to have similar experiences and exposures, thereby, forming more homogeneous comparison groups. While no significant differences were observed in samples from farms, male birds in LBMs were significantly more likely to test positive for IAV than female birds (49.1% vs. 44.1%, respectively; *p*  > 0.01). Across all three habitats, H5 positivity appeared to be associated with male birds.

Furthermore, the species stratum suggested that duck samples might be the primary contributor to sex differences in IAV (54.9% in males vs. 37.3% in females; *p*  > 0.0001) and H5 positivity (30.6% in males vs. 2.8% in females; *p*  > 0.0001). Male quail samples also showed an association with IAV positivity. However, as these samples were all from one market (Market 2), they likely did not significantly impact the overall market sampling or other habitats.

To further investigate any potential biasing factors, we performed a double stratification based on species type (Table 3). Interestingly, when we stratified on multiple variables, much of the significance for sex differences in chickens disappeared, with only farm chickens showing any kind of male positivity bias, and the opposite trend in juvenile female free-range chicken. The increased male positivity observation for ducks remained. Male ducks had significantly higher positivity rates in every stratification category in which there was data in our dataset. This pattern in both ducks and chickens held true for H5 positivity as well.

### 3.3. Bird Age and IAV Positivity

Our analysis found that birds that had been classified as “AHY" had significantly higher influenza positivity rates compared to birds that had been classified as “HY" (41.9% vs. 34.5%, respectively; *p*  < 0.0001; Table 1). This did not hold true for H5 positivity (5.5% vs. 7.1%, respectively; *p*  < 0.001). We stratified the samples in a similar manner as the bird sex association analysis to determine any sampling bias that could account for the unexpected finding. The results are shown in Table 4.

The results in the “sample type” and “location” strata for both IAV and H5 positivity were mixed, as was observed in the bird sex and influenza positivity stratification. However, AHY birds had higher IAV positivity rates in both free-range duck farms (39.0% vs. 35.6%, respectively; *p*  < 0.01) and LBM (58.8% vs. 46.3%, respectively; *p*  < 0.0001) strata. Unlike the results for bird sex associations, ducks had no significant differences between age groups, whereas chickens did show a significantly higher proportion of AHY birds with IAV positivity compared to HY (61.1% vs. 42.0%, respectively; *p*  < 0.0001).

We also found no age positivity differences in female birds, but male AHY birds had significantly higher IAV positivity rates compared to HY (60.2% vs. 44.8%, respectively; *p*  < 0.0001). There were no age-IAV positivity differences among different health status stratum. Furthermore, two categories showed higher H5 rates in AHY birds after stratification: LBM (30.5% vs. 12.2%, respectively; *p*  > 0.0001) samples and male bird (28.8% vs. 15.1%, respectively; *p*  > 0.0001) samples. Sick birds also showed higher H5 positivity in the AHY classification (11.1% vs. 3.2%, respectively; *p*  > 0.0001).

Since we found significant associations between bird sex and IAV positivity, we used double stratification on relevant variables and bird sex to eliminate any bias originating from the sex of the bird on the relationship between age classification and IAV positivity. The results of this double stratification are shown in Table 5. The pattern of higher rates of IAV positive in AHY birds remained for most of the strata even when secondarily stratified by sex. However, more of the male strata (7/8) in the various categories showed stronger AHY IAV positivity associations than the female stratified categories (4/8). Interestingly, in every H5 category except for one (female free-range farm birds) that had significantly different positivity rates in the double stratification analysis, AHY birds had higher H5 positivity.

**Figure 1:**
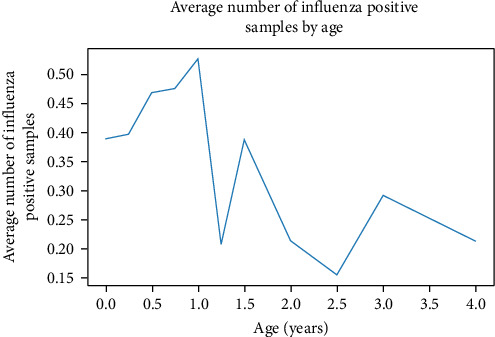
Influenza A positivity by age of birds. IAV positivity is displayed for avian samples grouped by age rounded to quarter year increments. IAV, influenza A virus.

To visualize the relationship between age and IAV positivity, samples were grouped by age in quarter year increments and the average IAV positivity was calculated for each age group, as shown in Figure 1. There is an initial upward trend in IAV positivity rate that peaks around 1 year (12–14 months) followed by a general fluctuating downward trend as age increases. It should be noted that birds in the 12–14 month peak are classified as AHY.

### 3.4. qRT-PCR Ct Values as Viral Burden

**Figure 2:**
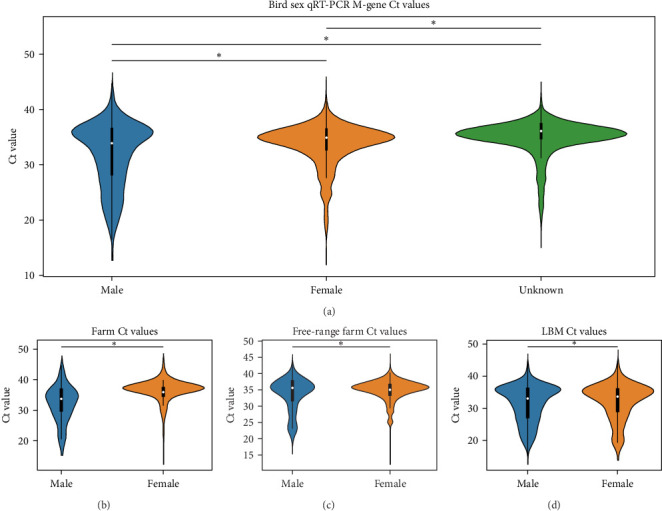
Violin plots of qRT-PCR Ct values for bird sexes. Violin plots illustrating the distribution of qRT-PCR M-gene Ct values between bird sexes in (a) the entire dataset and in specific habitats classified as (b) farm, (c) free-range farm, and (d) LBMs. The color-coding represents chicken (blue), duck (orange), quail (green), and environmental (feces) samples (red). Asterisks indicate significant differences (*α* = 0.05) between the indicated distributions as determined by Kolmogorov–Smirnov two-sample tests. Ct, cycle threshold; LBMs, live bird markets; M, matrix; qRT-PCR, reverse transcriptase polymerase chain reaction.

**Figure 3:**
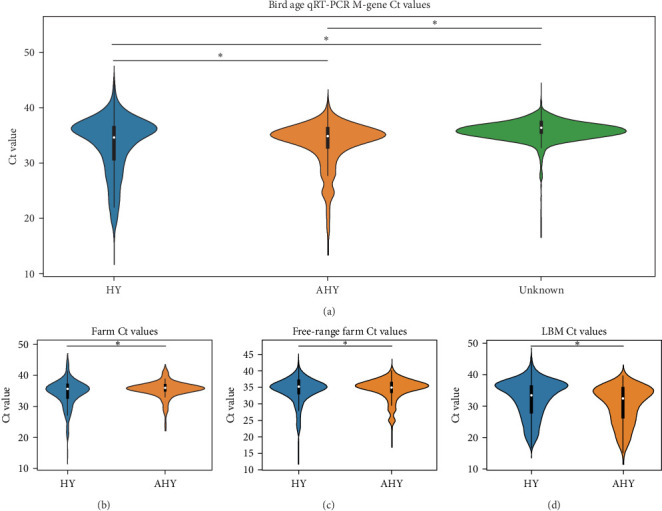
Violin plots of qRT-PCR Ct values for sample ages. Violin plots illustrating the distribution of qRT-PCR M-gene Ct values across different age classifications in (a) the entire dataset and in specific habitats classified as (b) farm, (c) free-range farm, and (d) LBMs. The color-coding represents samples from birds in their HY (blue), after their HY (orange), and unknown ages (green). Asterisks indicate significant differences (*α* = 0.05) between the indicated distributions as determined by Kolmogorov–Smirnov two-sample tests. AHY, after hatch year; Ct, cycle threshold; HY, hatch year; LBMs, live bird markets; M, matrix; qRT-PCR, reverse transcriptase polymerase chain reaction.

We compared the distributions of Ct values in three different categories to determine differences in viral loads among different hosts, sexes, and age classifications using Kolmogorov–Smirnov two-sample tests.  Figure 2 shows the Ct value distributions among sex classifications. Before stratification, male bird Ct values clustered lower than female birds, and both sexes were lower than unknown samples, which were largely environmental samples. This pattern held after stratification into different habitats, with farm birds and free-range farm birds showing clear differences in Ct values between male and female birds, and LBM samples showing a smaller, but still statistically significant difference between male and female birds. Similarly, Figure 3 shows the Ct value distribution among age classifications, with AHY farm samples and free-range farm samples clustering at higher Ct values compared to HY birds.

**Figure 4:**
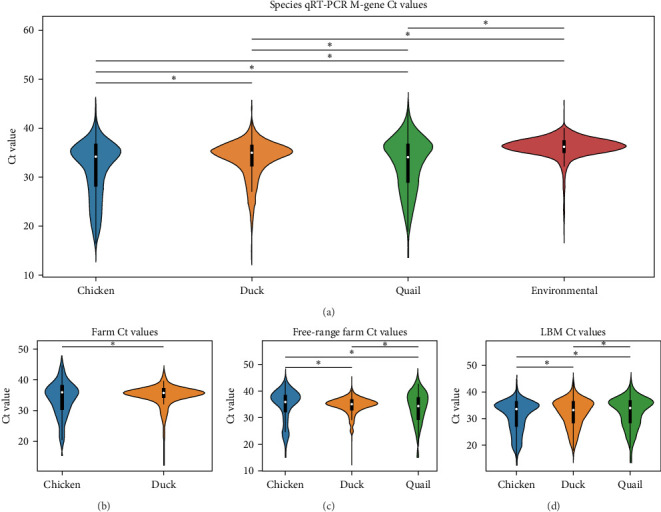
Violin plots of qRT-PCR Ct values for sample hosts. Violin plots illustrating the distribution of qRT-PCR M-gene Ct values across different host species and environmental samples in (a) the entire dataset and in specific habitats classified as (b) farm, (c) free-range farm, and (d) LBMs. The color-coding represents chicken (blue), duck (orange), quail (green), and environmental (feces) samples (red). Asterisks indicate significant differences (*α* = 0.05) between the indicated distributions as determined by Kolmogorov–Smirnov two-sample tests. Ct, cycle threshold; LBMs, live bird markets; M, matrix; qRT-PCR, reverse transcriptase polymerase chain reaction.

Finally, Figure 4 shows a similar Ct value analysis for different hosts in our sample, namely, chickens, ducks, quail, and environmental samples. Chicken and quail samples had Ct value distributions that clustered the lowest, followed by duck samples. Environmental samples had the highest Ct values (and thus, the lowest viral burdens). The pattern largely held for farm samples and free-range farm samples, but was almost lost in LBM samples, with duck sample clustering much lower in the LBM stratum compared to other strata.

## 4. Discussion

Active avian influenza surveillance is critical to our understanding of the distribution and dynamics of influenza viruses, informing both risk assessment models and pandemic preparedness plans [8]. Correlating metadata with risk of infection and viral load can highlight variables that play important roles in influenza transmission and suggest possible mitigation strategies for higher risk situations. During the period from late November 2016 through late November 2022, we conducted active avian influenza surveillance in Bangladesh, collecting samples from birds on farms, in markets, and in the wild and preformed qRT-PCR to determine both IAV and H5 infectivity status. Overall, 30.3% of our samples were positive for IAV and 4.9% were positive for H5. Unsurprisingly, the highest rates of influenza positivity were found in the LBMs. This trend was especially prominent for H5 positive samples, with market samples at 13.7% positive vs. farm samples at 1.3% positive. Chicken and quail had the highest rates of IAV positivity, followed closely by ducks. Environmental samples had the lowest IAV positive.

Two categories emerged with notable correlations to IAV positivity in our initial analysis: sex and age. In the complete dataset, both IAV and H5 positivity rates were significantly higher in male birds compared to female birds. This male bias persisted even after stratification. Further analysis through double stratification identified ducks as the primary contributors to this pattern, though the lack of significant differences in chickens may be attributed to the reduced statistical power resulting from the smaller sample size in double stratification. Among avian surveillance studies that have reported on sex bias in ducks, the results seem to be mixed, with some groups finding a male bias in IAV prevalence [37, 38] and other groups finding no evidence of sex bias [39, 40]. Different mechanisms have been proposed to explain this bias, from behavioral (e.g., foraging and aggression) to biological (e.g., testosterone/estrogen suppressing or enhancing the immune system, respectively) [41]. A behavioral mechanism seems plausible for the dataset analyzed here, especially as free-range farm ducks have few restrictions that would disallow sex-specific behavioral differences while foraging (e.g., perhaps male ducks are more likely to interact with other ducks while foraging).

Furthermore, this effect could be specific to the farms and markets that were sampled in Bangladesh, such as caging and farming preferences that might put male birds at higher risk. For example, female ducks, favored for their egg-laying abilities, are typically kept longer in Bangladesh. As these ducks age and cease laying eggs, they are sold for meat. Conversely, male ducks are more commonly sold for meat from the outset. This practice results in a higher number of males in the market, potentially leading to a sampling bias regarding sex. The phenomenon could also be driven by duck behavior rather than a biological difference. When the source of LBM birds was considered, the male/female IAV positivity disparity was dramatically seen in birds that came from backyard farms (55.2% male vs. 28.4% female; *p*  < 0.0001) and was absent in birds sourced from commercial farms (57.9% male vs. 50.0% female; *p*  > 0.05). As mentioned above, this could suggest that male duck behavior on backyard farms, where there is poor biosecurity, is the primary contributor to this phenomenon. More studies are needed to validate this finding. However, should it hold true, male infectivity bias would be an important factor to consider in pandemic prediction and risk assessment models.

The correlation of age and IAV positivity was less robust in our dataset compared to sex correlations. However, this correlation was interesting because it stands in tension with the conventional wisdom that juvenile birds are more susceptible to influenza infection compared to adult birds [42, 43]. Accordingly, there were mixed results when the data was stratified, with AHY birds showing higher IAV positivity rates in some stratum, but lower or nonsignificant differences in others. Furthermore, H5 positivity rates were largely as expected in terms of bird age, though it was interesting that AHY birds in the market had higher H5 positivity rates than HY birds. There is likely market bias here, as there is much more H5 pressure in the market in general and there may be an age preference that buyers and sellers have for market birds. When we looked beyond age classification to actual age, we found that the highest infection rates were among birds that were 1 year old, which is just after the cutoff for HY and AHY classification, possibly giving an unrealistic perception from the age classification category. Thus, juvenile birds show higher IAV positivity rate compared to adult birds with a peak just after 1 year, which drives the higher IAV positivity in AHY birds compared to HY birds. This explanation is supported by the general downward trend that appears when infection rates are plotted against bird age from 0 to 4 years. This highlights the need for strategic variable decisions when incorporating metadata into risk assessment models [8].

Beyond IAV positivity rates, viral burden and shedding also play crucial roles in influenza transmission and are important factors to consider in risk assessment models. In our dataset, chicken samples had significantly higher Ct values compared to ducks and environmental samples, suggesting that chickens might pose a more significant risk for zoonotic influenza transmission. We also found that male birds and HY birds had lower Ct values. Interestingly, considering the previous finding that birds classified as AHY had a higher IAV positivity rate, HY birds appeared to have a higher viral load compared to AHY and unknown samples. This held true for farm samples and free-range farm samples, but the pattern inverted in LBM samples. This could be a contributing factor to the unexpected finding that AHY birds had a higher IAV positivity rate.

Each of these patterns held true across habitat strata, but it should be noted that for each of these categories, the LBM stratum largely erased the difference in distributions (though not completely), with all distributions in the subcategories (chicken/duck/quail, male/female, and HY/AHY) clustering lower than in the farm or free-range farm habitats. This is probably due to the high market pressure of IAV and H5, which changes the dynamics of transmission and susceptibility. Wild birds were sampled using environmental methods (fecal sampling), which resulted in the highest Ct values compared to farm or market samples. However, this difference is likely due to the sampling approach rather than a true difference in influenza prevalence among wild birds in Bangladesh. Fresh samples taken directly from an infected bird typically yield higher viral loads, whereas fecal samples, which may have been exposed to environmental conditions for an unknown period, tend to show lower viral loads.

Overall, these results provide important insights into influenza dynamics and transmission in domestic and wild birds in Bangladesh. As active surveillance resumes, it will be important to continue to monitor these trends and incorporate data into larger risk assessment and pandemic prediction and preparation models.

## Figures and Tables

**Table 1:** Active avian influenza surveillance in wild and domestic birds in Bangladesh from 2017 to 2022.

Variable	Collected Samples, no. (%)	Influenza A positive, no. (%)	*p*-value	Significance	H5 positive, no. (%)	*p*-value	Significance
Sample type							
**All**	**20,069 (100.0%)**	**6082 (30.3%)**	**<0.0001**	*⁣* ^*∗∗∗∗*^	**976 (4.9%)**	**<0.0001**	*⁣* ^*∗∗∗∗*^
Cloacal	1508 (7.5%)	845 (56.0%)	Ref	—	129 (8.6%)	Ref	—
Oral–pharyngeal swab	8776 (43.7%)	3537 (40.3%)	<0.0001	*⁣* ^*∗∗∗∗*^	687 (7.8%)	>0.05	ns
Combined oral and cloacal	452 (2.3%)	41 (9.1%)	<0.0001	*⁣* ^*∗∗∗∗*^	2 (0.4%)	<0.0001	*⁣* ^*∗∗∗∗*^
Water	1605 (8.0%)	705 (43.9%)	<0.0001	*⁣* ^*∗∗∗∗*^	157 (9.8%)	>0.05	ns
Environmental	7728 (38.5%)	954 (12.3%)	<0.0001	*⁣* ^*∗∗∗∗*^	1 (0.0%)	<0.0001	*⁣* ^*∗∗∗∗*^
**Habitat**	**Overall**		**<0.0001**	*⁣* ^*∗∗∗∗*^	—	**<0.0001**	*⁣* ^*∗∗∗∗*^
Free-range farm	6174 (30.8%)	2256 (36.5%)	Ref	—	186 (3.0%)	Ref	—
Farm	689 (3.4%)	292 (42.4%)	<0.01	*⁣* ^*∗∗*^	46 (6.7%)	<0.0001	*⁣* ^*∗∗∗∗*^
LBM	5425 (27.0%)	2568 (47.3%)	<0.0001	*⁣* ^*∗∗∗∗*^	743 (13.7%)	<0.0001	*⁣* ^*∗∗∗∗*^
Migrating	281 (1.4%)	30 (10.7%)	<0.0001	*⁣* ^*∗∗∗∗*^	1 (0.4%)	<0.05	*⁣* ^*∗*^
Nonmigrating	3 (0.0%)	0 (0.0%)	>0.05	ns	0 (0.0%)	>0.05	ns
Unknown	7497 (37.4%)	938 (12.5%)	<0.0001	*⁣* ^*∗∗∗∗*^	0 (0.0%)	<0.0001	*⁣* ^*∗∗∗∗*^
**Source of Bird (LBM)^a^**	**Overall**		**<0.0001**	*⁣* ^*∗∗∗∗*^	—	**<0.0001**	*⁣* ^*∗∗∗∗*^
Backyard farm	1836 (33.8%)	980 (53.4%)	<0.0001	*⁣* ^*∗∗∗∗*^	430 (23.4%)	<0.0001	*⁣* ^*∗∗∗∗*^
Commercial farm	3589 (66.2%)	1588 (44.2%)	<0.0001	*⁣* ^*∗∗∗∗*^	313 (8.7%)	<0.0001	*⁣* ^*∗∗∗∗*^
**Age**	**Overall**		**<0.0001**	*⁣* ^*∗∗∗∗*^	—	**<0.0001**	*⁣* ^*∗∗∗∗*^
HY	10,966 (54.6%)	3784 (34.5%)	Ref	—	775 (7.1%)	Ref	—
AHY	3657 (18.2%)	1531 (41.9%)	<0.0001	*⁣* ^*∗∗∗∗*^	200 (5.5%)	<0.001	*⁣* ^*∗∗∗*^
Unknown	5446 (27.1%)	767 (14.1%)	<0.0001	*⁣* ^*∗∗∗∗*^	1 (0.0%)	<0.0001	*⁣* ^*∗∗∗∗*^
**Sex^b^**	**Overall**		**<0.0001**	*⁣* ^*∗∗∗∗*^	—	**<0.0001**	*⁣* ^*∗∗∗∗*^
Female	7257 (36.2%)	2770 (38.2%)	Ref	—	258 (3.6%)	<0.0001	*⁣* ^*∗∗∗∗*^
Male	4156 (20.7%)	1933 (46.5%)	<0.0001	*⁣* ^*∗∗∗∗*^	685 (16.5%)	<0.0001	*⁣* ^*∗∗∗∗*^
Unknown	8656 (43.1%)	1379 (15.9%)	<0.0001	*⁣* ^*∗∗∗∗*^	33 (0.4%)	<0.0001	*⁣* ^*∗∗∗∗*^
**Sex (LBM)^a^**	**Overall**		**<0.01**	*⁣* ^*∗∗*^	—	**<0.0001**	*⁣* ^*∗∗∗∗*^
Female	1689 (35.7%)	745 (44.1%)	—	—	182 (10.8%)	—	—
Male	3036 (64.3%)	1491 (49.1%)	—	—	539 (17.8%)	—	—
**Species**	**Overall**		**<0.0001**	*⁣* ^*∗∗∗∗*^	—	**<0.0001**	*⁣* ^*∗∗∗∗*^
Duck	7527 (37.5%)	2978 (39.6%)	Ref	—	604 (8.0%)	Ref	—
Chicken	4058 (20.2%)	1734 (42.7%)	<0.01	*⁣* ^*∗∗*^	340 (8.4%)	>0.05	ns
Quail	1000 (5.0%)	439 (43.9%)	<0.01	*⁣* ^*∗∗*^	32 (3.2%)	<0.0001	*⁣* ^*∗∗∗∗*^
Environmental	7484 (37.3%)	931 (12.4%)	<0.0001	*⁣* ^*∗∗∗∗*^	0 (0.0%)	<0.0001	*⁣* ^*∗∗∗∗*^
**Location**	**Overall**		**<0.0001**	*⁣* ^*∗∗∗∗*^	—	**<0.0001**	*⁣* ^*∗∗∗∗*^
Market 1	1815 (9.0%)	692 (38.1%)	Ref	—	181 (10.0%)	Ref	—
Market 2	1000 (5.0%)	439 (43.9%)	<0.01	*⁣* ^*∗∗*^	32 (3.2%)	<0.0001	*⁣* ^*∗∗∗∗*^
Farm 1	700 (3.5%)	215 (30.7%)	<0.0001	*⁣* ^*∗∗∗∗*^	4 (0.6%)	<0.0001	*⁣* ^*∗∗∗∗*^
Market 3	1800 (9.0%)	892 (49.6%)	<0.0001	*⁣* ^*∗∗∗∗*^	325 (18.1%)	<0.0001	*⁣* ^*∗∗∗∗*^
Market 4	1778 (8.9%)	1005 (56.5%)	<0.0001	*⁣* ^*∗∗∗∗*^	348 (19.6%)	<0.0001	*⁣* ^*∗∗∗∗*^
Tanguar Haor	11,671 (58.2%)	2609 (22.4%)	<0.0001	*⁣* ^*∗∗∗∗*^	14 (0.1%)	<0.0001	*⁣* ^*∗∗∗∗*^
Farm 2	360 (1.8%)	101 (28.1%)	<0.001	*⁣* ^*∗∗∗*^	50 (13.9%)	<0.05	*⁣* ^*∗*^
Lake	600 (3.0%)	48 (8.0%)	<0.0001	*⁣* ^*∗∗∗∗*^	4 (0.7%)	<0.0001	*⁣* ^*∗∗∗∗*^
Farm 3	345 (1.7%)	83 (24.1%)	<0.0001	*⁣* ^*∗∗∗∗*^	18 (5.2%)	<0.01	*⁣* ^*∗∗*^
**Health status**	**Overall**		**<0.0001**	*⁣* ^*∗∗∗∗*^	—	**<0.0001**	*⁣* ^*∗∗∗∗*^
Healthy	12,035 (60.0%)	5051 (42.0%)	Ref	—	959 (8.0%)	Ref	—
Sick	206 (1.0%)	48 (23.3%)	<0.0001	*⁣* ^*∗∗∗∗*^	8 (3.9%)	<0.0001	*⁣* ^*∗∗∗∗*^
Dead	52 (0.3%)	22 (42.3%)	>0.05	ns	8 (15.4%)	>0.05	ns
Undetermined	7776 (38.7%)	961 (12.4%)	<0.0001	*⁣* ^*∗∗∗∗*^	1 (0.0%)	<0.0001	*⁣* ^*∗∗∗∗*^

*Note:* The bold indicates that the analysis was for the whole group, separate from the individual analysis of sub-group categories relative to the indicated reference that are below the bold values.

Abbreviations: AHY, after hatch year; HY, hatch year; LBM, live bird market; ns, not significant.

^a^Categories marked “LBM” include only samples taken from live bird markets.

^b^Water samples associated with a sex were retained in the analysis (male: *n* = 673 and female: *n* = 712).

*⁣* ^*∗*^, *p*  < 0.05; *⁣*^*∗∗*^, *p*  < 0.01; *⁣*^*∗∗∗*^, *p*  < 0.001; *⁣*^*∗∗∗∗*^, *p*  < 0.0001.

**Table 2:** Sex stratification of variables for years 2017–2022.

Variable	Total samples^a^, no. (%)			Influenza A positive, no. (% positive per sex)				H5 positive, no. (% positive per sex)			
	*n*	Female	Male	Female	Male	*p*-value	Significance	Female	Male	*p*-value	Significance
**Sample type**	**11,413**	—	—	—	—	—	—	—	—	—	—
Cloacal	1503 (13.2%)	1041 (69.3%)	462 (30.7%)	629 (60.4%)	213 (46.1%)	<0.0001	*⁣* ^*∗∗∗∗*^	39 (3.7%)	89 (19.3%)	<0.0001	*⁣* ^*∗∗∗∗*^
Oral–pharyngeal swab	449 (3.9%)	399 (88.9%)	50 (11.1%)	27 (6.8%)	14 (28.0%)	<0.0001	*⁣* ^*∗∗∗∗*^	2 (0.4%)	0 (0.0%)	>0.05	ns
Combined oral and cloacal	8076 (70.8%)	5105 (63.2%)	2971 (36.8%)	1783 (34.9%)	1435 (48.3%)	<0.0001	*⁣* ^*∗∗∗∗*^	145 (2.8%)	514 (17.3%)	<0.0001	*⁣* ^*∗∗∗∗*^
Water	1385 (12.1%)	712 (51.4%)	673 (48.6%)	331 (46.5%)	271 (40.3%)	<0.05	*⁣* ^*∗*^	72 (10.1%)	82 (12.2%)	>0.05	ns
**Habitat**	**11,398**	—	—	—	—	—	—	—	—	—	—
Farm	689 (6.0%)	509 (73.9%)	180 (26.1%)	215 (42.2%)	77 (42.8%)	>0.05	ns	0 (0.0%)	46 (25.6%)	<0.0001	*⁣* ^*∗∗∗∗*^
Free-range farm	5984 (52.5%)	5053 (84.4%)	931 (15.6%)	1809 (35.8%)	359 (38.6%)	>0.05	ns	76 (1.5%)	100 (10.7%)	<0.0001	*⁣* ^*∗∗∗∗*^
LBM	4725 (41.5%)	1689 (35.7%)	3036 (64.3%)	745 (44.1%)	1491 (49.1%)	<0.01	*⁣* ^*∗∗*^	182 (10.8%)	539 (17.8%)	<0.0001	*⁣* ^*∗∗∗∗*^
**Source of bird (LBM)^a^**	**6647**	—	—	—	—	—	—	—	—	—	—
Backyard farm	2677 (40.3%)	995 (37.2%)	1682 (62.8%)	310 (31.2%)	966 (57.4%)	<0.0001	*⁣* ^*∗∗∗∗*^	127 (12.8%)	422 (25.1%)	<0.0001	*⁣* ^*∗∗∗∗*^
Commercial farm	3850 (57.9%)	1436 (37.3%)	2414 (62.7%)	593 (41.3%)	943 (39.1%)	>0.05	ns	113 (7.9%)	263 (10.9%)	<0.01	*⁣* ^*∗∗*^
Hatchery	120 (1.8%)	93 (77.5%)	27 (22.5%)	9 (9.7%)	7 (25.9%)	>0.05	ns	0 (0.0%)	0 (0.0%)	N/A	N/A
**Age**	**11,248**	—	—	—	—	—	—	—	—	—	—
HY	7591 (67.5%)	3904 (51.4%)	3687 (48.6%)	1504 (38.5%)	1650 (44.8%)	<0.0001	*⁣* ^*∗∗∗∗*^	188 (4.8%)	555 (15.1%)	<0.0001	*⁣* ^*∗∗∗∗*^
AHY	3657 (32.5%)	3197 (87.4%)	460 (12.6%)	1254 (39.2%)	277 (60.2%)	<0.0001	*⁣* ^*∗∗∗∗*^	70 (2.2%)	130 (28.3%)	<0.0001	*⁣* ^*∗∗∗∗*^
**Species**	**11,413**	—	—	—	—	—	—	—	—	—	—
Duck	7230 (63.3%)	5799 (80.2%)	1431 (19.8%)	2162 (37.3%)	786 (54.9%)	<0.0001	*⁣* ^*∗∗∗∗*^	165 (2.8%)	438 (30.6%)	<0.0001	*⁣* ^*∗∗∗∗*^
Chicken	4058 (35.6%)	1358 (33.5%)	2700 (66.5%)	597 (44.0%)	1137 (42.1%)	>0.05	ns	93 (6.8%)	247 (9.1%)	<0.05	*⁣* ^*∗*^
Quail	125 (1.1%)	100 (80.0%)	25 (20.0%)	11 (11.0%)	10 (40.0%)	<0.01	*⁣* ^*∗∗*^	0 (0.0%)	0 (0.0%)	N/A	N/A
**Location**	**11,413**	—	—	—	—	—	—	—	—	—	—
Market 1	1815 (15.9%)	464 (25.6%)	1351 (74.4%)	166 (35.8%)	526 (38.9%)	>0.05	ns	35 (7.5%)	146 (10.8%)	>0.05	ns
Market 2^b^	125 (6.9%)	100 (80.0%)	25 (20.0%)	11 (11.0%)	10 (40.0%)	<0.01	*⁣* ^*∗∗*^	0 (0.0%)	0 (0.0%)	N/A	N/A
Farm 1	700 (6.1%)	697 (99.6%)	3 (0.4%)	213 (30.6%)	0 (0.0%)	>0.05	ns	4 (0.6%)	0 (0.0%)	>0.05	ns
Market 3	1800 (15.8%)	657 (36.5%)	1143 (63.5%)	353 (53.7%)	539 (47.2%)	<0.01	*⁣* ^*∗∗*^	106 (16.1%)	219 (19.2%)	>0.05	ns
Market 4	1778 (15.6%)	597 (33.6%)	1181 (66.4%)	292 (48.9%)	713 (60.4%)	<0.0001	*⁣* ^*∗∗∗∗*^	84 (14.1%)	264 (22.4%)	<0.0001	*⁣* ^*∗∗∗∗*^
Tanguar Haor	4186 (36.7%)	4129 (98.6%)	57 (1.4%)	1654 (40.1%)	24 (42.1%)	>0.05	ns	14 (0.3%)	0 (0.0%)	>0.05	ns
Farm 2	360 (3.2%)	74 (20.6%)	286 (79.4%)	19 (25.7%)	82 (28.7%)	>0.05	ns	8 (10.8%)	42 (14.7%)	>0.05	ns
Lake	304 (2.7%)	290 (95.4%)	14 (4.6%)	17 (5.9%)	1 (7.1%)	>0.05	ns	3 (1.0%)	0 (0.0%)	>0.05	ns
Farm 3	345 (3.0%)	249 (72.2%)	96 (27.8%)	45 (18.1%)	38 (39.6%)	<0.0001	*⁣* ^*∗∗∗∗*^	4 (1.6%)	14 (14.6%)	<0.0001	*⁣* ^*∗∗∗∗*^
**Health status** *⁣* ^*∗*^	**11,288**	—	—	—	—	—	—	—	—	—	—
Healthy	11,045 (97.8%)	6980 (63.2%)	4065 (36.8%)	2724 (39.0%)	1891 (46.5%)	<0.0001	*⁣* ^*∗∗∗∗*^	257 (3.7%)	670 (16.5%)	<0.0001	*⁣* ^*∗∗∗∗*^
Sick	204 (1.8%)	173 (84.8%)	31 (15.2%)	32 (18.5%)	14 (45.2%)	<0.01	*⁣* ^*∗∗*^	1 (0.6%)	7 (22.6%)	<0.0001	*⁣* ^*∗∗∗∗*^
Dead	39 (0.3%)	4 (10.3%)	35 (89.7%)	3 (75.0%)	18 (51.4%)	>0.05	ns	0 (0.0%)	8 (22.9%)	>0.05	ns

*Note:* The bold indicates that the analysis was for the whole group, separate from the individual analysis of sub-group categories relative to the indicated reference that are below the bold values.

Abbreviations: AHY, after hatch year; HY, hatch year; LBM, live bird market; ns, not significant.

^a^Categories marked “LBM” include only samples taken from live bird markets.

^b^The samples from Market 2 market were all quail.

*⁣* ^*∗*^,  *p*  < 0.05;  ^*∗∗*^, *p*  < 0.01; *⁣*^*∗∗∗*^,  *p*  < 0.001; *⁣*^*∗∗∗∗*^, *p*  < 0.0001.

**Table 3:** Sex double and triple stratification of important variables for years 2017–2022.

Variable	Total samples^a^, no. (%)			Influenza A positive, no. (% positive per sex)				H5 positive, no. (% positive per sex)			
	*n*	Female	Male	Female	Male	*p*-value	Significance	Female	Male	*p*-value	Significance
**Habitat: farm**	**689**	—	—	—	—	—	—	—	—	—	—
Duck	419 (60.8%)	419 (100.0%)	0 (0.0%)	211 (50.4%)	N/A	N/A	N/A	0 (0.0%)	N/A	N/A	N/A
Chicken	270 (39.2%)	90 (33.3%)	180 (66.7%)	4 (4.4%)	77 (42.8%)	<0.0001	*⁣* ^*∗∗∗∗*^	0 (0.0%)	46 (25.6%)	<0.0001	*⁣* ^*∗∗∗∗*^
**Habitat: free-range farm**	**5984**	—	—	—	—	—	—	—	—	—	—
Duck	5174 (86.5%)	4851 (93.8%)	323 (6.2%)	1728 (35.6%)	163 (50.5%)	<0.0001	*⁣* ^*∗∗∗∗*^	58 (1.2%)	61 (18.9%)	<0.0001	*⁣* ^*∗∗∗∗*^
Chicken	810 (13.5%)	202 (24.9%)	608 (75.1%)	81 (40.1%)	196 (32.2%)	>0.05	ns	18 (2.2%)	39 (6.4%)	>0.05	ns
**Habitat: LBM**	**4600**	—	—	—	—	—	—	—	—	—	—
Duck	1622 (35.3%)	523 (32.2%)	1099 (67.8%)	222 (42.4%)	617 (56.1%)	<0.0001	*⁣* ^*∗∗∗∗*^	107 (20.5%)	377 (34.3%)	<0.0001	*⁣* ^*∗∗∗∗*^
Chicken	2978 (64.7%)	1066 (35.8%)	1912 (64.2%)	512 (48.0%)	864 (45.2%)	>0.05	ns	75 (2.5%)	162 (8.5%)	>0.05	ns
**Farm chickens^a^**	**270**	—	—	—	—	—	—	—	—	—	—
Age: HY	270 (100.0%)	90 (33.3%)	180 (66.7%)	4 (4.4%)	77 (42.8%)	<0.0001	*⁣* ^*∗∗∗∗*^	0 (0.0%)	46 (25.6%)	<0.0001	*⁣* ^*∗∗∗∗*^
**Free-range farm ducks**	**5030**	—	—	—	—	—	—	—	—	—	—
Age: HY	2084 (41.4%)	1877 (90.1%)	207 (9.9%)	641 (34.2%)	99 (47.8%)	<0.001	*⁣* ^*∗∗∗*^	29 (1.5%)	29 (14.0%)	<0.0001	*⁣* ^*∗∗∗∗*^
Age: AHY	2946 (58.6%)	2830 (96.1%)	116 (3.9%)	1078 (38.1%)	64 (55.2%)	<0.001	*⁣* ^*∗∗∗*^	29 (1.0%)	32 (27.6%)	<0.0001	*⁣* ^*∗∗∗∗*^
**Free-range farm chickens**	**810**	—	—	—	—	—	—	—	—	—	—
Age: HY	750 (92.6%)	202 (26.9%)	548 (73.1%)	81 (40.1%)	165 (30.1%)	<0.05	*⁣* ^*∗*^	18 (8.9%)	35 (6.4%)	>0.05	ns
Age: AHY	60 (7.4%)	0 (0.0%)	60 (100.0%)	N/A	31 (51.7%)	N/A	N/A	N/A	4 (6.7%)	N/A	N/A
**Source: backyard farm (LBM)^b^**	**2677**	—	—	—	—	—	—	—	—	—	—
Duck	2059 (76.9%)	885 (43.0%)	1174 (57.0%)	251 (28.4%)	648 (55.2%)	<0.0001	*⁣* ^*∗∗∗∗*^	122 (13.8%)	359 (30.6%)	<0.0001	*⁣* ^*∗∗∗∗*^
Chicken	618 (23.1%)	110 (17.8%)	508 (82.2%)	59 (53.6%)	318 (62.6%)	<0.0001	*⁣* ^*∗∗∗∗*^	N/A	63 (12.4%)	<0.05	*⁣* ^*∗*^
**Source: commercial farm (LBM)^b^**	**3725**	—	—	—	—	—	—	—	—	—	—
Duck	285 (7.7%)	88 (30.9%)	197 (69.1%)	44 (50.0%)	114 (57.9%)	>0.05	ns	25 (28.4%)	79 (40.1%)	>0.05	ns
Chicken	3440 (92.3%)	1248 (36.3%)	2192 (63.7%)	538 (43.1%)	819 (37.4%)	<0.01	*⁣* ^*∗∗*^	88 (7.1%)	184 (8.4%)	>0.05	ns
**LBM ducks**	**1622**	—	—	—	—	—	—	—	—	—	—
Age: HY	1282 (79.0%)	398 (31.0%)	884 (69.0%)	166 (41.7%)	481 (54.4%)	<0.0001	*⁣* ^*∗∗∗∗*^	68 (17.1%)	291 (32.9%)	<0.0001	*⁣* ^*∗∗∗∗*^
Age: AHY	340 (21.0%)	125 (36.8%)	215 (63.2%)	56 (44.8%)	136 (63.3%)	<0.01	*⁣* ^*∗∗*^	39 (31.2%)	86 (40.0%)	>0.05	ns
**LBM chickens**	**2978**	—	—	—	—	—	—	—	—	—	—
Age: HY	2876 (96.6%)	1033 (35.9%)	1843 (64.1%)	490 (47.4%)	818 (44.4%)	>0.05	ns	73 (7.1%)	154 (8.4%)	>0.05	ns
Age: AHY	102 (3.4%)	33 (32.4%)	69 (67.6%)	22 (66.7%)	46 (66.7%)	>0.05	ns	2 (6.1%)	8 (11.6%)	>0.05	ns

*Note:* The bold indicates that the analysis was for the whole group, separate from the individual analysis of sub-group categories relative to the indicated reference that are below the bold values.

Abbreviations: AHY, after hatch year; HY, hatch year; LBM, live bird market; ns, not significant.

^a^No AHY samples were taken on farms, thus, this data is the same as “Habitat: farm, chickens”.

^b^Categories marked “LBM” include only samples taken from live bird markets.

*⁣* ^*∗*^, *p*  < 0.05; *⁣*^*∗∗*^, *p*  < 0.01; *⁣*^*∗∗∗*^, *p*  < 0.001; *⁣*^*∗∗∗∗*^, *p*  < 0.0001.

**Table 4:** Age stratification of variables for years 2017–2022.

Variable	Total samples^a^, no. (%)			Influenza A positive, no. (% positive per age)				H5 positive, no. (% positive per age)			
	*n*	HY	AHY	HY	AHY	*p*-value	Significance	HY	AHY	*p*-value	Significance
**Sample type**	**12,123**	—	—	—	—	—	—	—	—	—	—
Cloacal	1503 (12.4%)	765 (50.9%)	738 (49.1%)	425 (55.6%)	415 (56.2%)	>0.05	ns	93 (12.2%)	36 (4.9%)	<0.0001	*⁣* ^*∗∗∗∗*^
Oral–pharyngeal swab^b^	8631 (71.2%)	5742 (66.5%)	2889 (33.5%)	2431 (42.3%)	1100 (38.1%)	<0.001	*⁣* ^*∗∗∗*^	529 (9.2%)	158 (5.5%)	<0.0001	*⁣* ^*∗∗∗∗*^
Combined oral and cloacal	434 (3.6%)	424 (97.7%)	10 (2.3%)	27 (6.4%)	7 (70.0%)	<0.0001	*⁣* ^*∗∗∗∗*^	0 (0.0%)	2 (20.0%)	>0.05	ns
Water	1555 (12.8%)	1535 (98.7%)	20 (1.3%)	689 (44.9%)	9 (45.0%)	>0.05	ns	153 (10.0%)	4 (20.0%)	>0.05	ns
**Habitat**	**14,623**	—	—	—	—	—	—	—	—	—	—
Farm	689 (4.7%)	476 (69.1%)	213 (30.9%)	192 (40.3%)	100 (46.9%)	>0.05	ns	46 (9.7%)	0 (0.0%)	<0.0001	*⁣* ^*∗∗∗∗*^
Free-range farm	6009 (41.1%)	3007 (50.0%)	3002 (50.0%)	1072 (35.7%)	1171 (39.0%)	<0.01	*⁣* ^*∗∗*^	121 (4.0%)	65 (2.2%)	<0.0001	*⁣* ^*∗∗∗∗*^
LBM	5425 (37.1%)	4983 (91.9%)	442 (8.1%)	2308 (46.3%)	260 (58.8%)	<0.0001	*⁣* ^*∗∗∗∗*^	608 (12.2%)	135 (30.5%)	<0.0001	*⁣* ^*∗∗∗∗*^
Unknown	2500 (17.1%)	2500 (100.0%)	0 (0.0%)	212 (8.5%)	N/A	N/A	N/A	0 (0.0%)	N/A	N/A	N/A
**Source of bird (LBM)^a^**	**7522**	—	—	—	—	—	—	—	—	—	—
Backyard farm	2677 (19.7%)	2080 (77.7%)	597 (22.3%)	958 (46.1%)	318 (53.3%)	<0.01	*⁣* ^*∗∗*^	409 (19.7%)	140 (23.5%)	<0.05	*⁣* ^*∗*^
Commercial farm	4725 (34.7%)	4635 (98.1%)	90 (1.9%)	1897 (40.9%)	57 (63.3%)	<0.0001	*⁣* ^*∗∗∗∗*^	364 (7.9%)	44 (48.9%)	<0.0001	*⁣* ^*∗∗∗∗*^
Hatchery	120 (0.9%)	120 (100.0%)	0 (0.0%)	16 (13.3%)	N/A	N/A	N/A	0 (0.0%)	N/A	N/A	N/A
**Sex**	**14,623**	—	—	—	—	—	—	—	—	—	—
Female	7101 (48.6%)	3904 (55.0%)	3197 (45.0%)	1504 (38.5%)	1254 (39.2%)	>0.05	ns	188 (4.8%)	70 (2.2%)	<0.0001	*⁣* ^*∗∗∗∗*^
Male	4147 (28.4%)	3687 (88.9%)	460 (11.1%)	1650 (44.8%)	277 (60.2%)	<0.0001	*⁣* ^*∗∗∗∗*^	555 (15.1%)	130 (28.3%)	<0.0001	*⁣* ^*∗∗∗∗*^
Unknown	3375 (23.1%)	3375 (100.0%)	0 (0.0%)	630 (18.7%)	0 (0.0%)	N/A	N/A	32 (0.9%)	0 (0.0%)	N/A	N/A
**Species**	**14,623**	—	—	—	—	—	—	—	—	—	—
Duck	7065 (48.3%)	3570 (50.5%)	3495 (49.5%)	1498 (42.0%)	1432 (41.0%)	>0.05	ns	417 (11.7%)	186 (5.3%)	<0.0001	*⁣* ^*∗∗∗∗*^
Chicken	4058 (27.8%)	3896 (96.0%)	162 (4.0%)	1635 (42.0%)	99 (61.1%)	<0.0001	*⁣* ^*∗∗∗∗*^	326 (8.4%)	14 (8.6%)	>0.05	ns
Quail	1000 (6.8%)	1000 (100.0%)	0 (0.0%)	436 (43.6%)	N/A	N/A	N/A	32 (3.2%)	N/A	N/A	N/A
Environmental (feces)	2500 (17.1%)	2500 (100.0%)	0 (0.0%)	212 (8.5%)	N/A	N/A	N/A	0 (0.0%)	N/A	N/A	N/A
**Location**	**13,623**	—	—	—	—	—	—	—	—	—	—
Market 1	1815 (13.3%)	1715 (94.5%)	100 (5.5%)	641 (37.4%)	51 (51.0%)	<0.01	*⁣* ^*∗∗*^	151 (8.8%)	30 (30.0%)	<0.0001	*⁣* ^*∗∗∗∗*^
Farm 1	550 (4.0%)	263 (47.8%)	287 (52.2%)	91 (34.6%)	111 (38.7%)	>0.05	ns	1 (0.4%)	3 (1.0%)	>0.05	ns
Market 3	1800 (13.2%)	1618 (89.9%)	182 (10.1%)	785 (48.5%)	107 (58.8%)	<0.05	*⁣* ^*∗*^	257 (15.9%)	68 (37.4%)	<0.0001	*⁣* ^*∗∗∗∗*^
Market 4	1778 (13.1%)	1423 (80.0%)	355 (20.0%)	795 (55.9%)	210 (59.2%)	>0.05	ns	264 (18.6%)	84 (23.7%)	<0.05	*⁣* ^*∗*^
Tanguar Haor	6671 (49.0%)	3988 (59.8%)	2683 (40.2%)	838 (21.0%)	1045 (38.9%)	<0.0001	*⁣* ^*∗∗∗∗*^	1 (0.0%)	13 (0.5%)	<0.001	*⁣* ^*∗∗∗*^
Farm 2	360 (2.6%)	335 (93.1%)	25 (6.9%)	101 (30.1%)	0 (0.0%)	<0.01	*⁣* ^*∗∗*^	50 (14.9%)	0 (0.0%)	>0.05	ns
Lake	304 (2.2%)	294 (96.7%)	10 (3.3%)	11 (3.7%)	7 (70.0%)	<0.0001	*⁣* ^*∗∗∗∗*^	1 (0.3%)	2 (20.0%)	<0.0001	*⁣* ^*∗∗∗∗*^
Farm 3	345 (2.5%)	330 (95.7%)	15 (4.3%)	83 (25.2%)	0 (0.0%)	>0.05	ns	18 (5.5%)	0 (0.0%)	>0.05	ns
**Health status**	**14,623**	—	—	—	—	—	—	—	—	—	—
Healthy	11,866 (81.1%)	8230 (69.4%)	3636 (30.6%)	3511 (42.7%)	1522 (41.9%)	>0.05	ns	761 (9.2%)	198 (5.4%)	<0.0001	*⁣* ^*∗∗∗∗*^
Sick	206 (1.4%)	188 (91.3%)	18 (8.7%)	42 (22.3%)	6 (33.3%)	>0.05	ns	6 (3.2%)	2 (11.1%)	>0.05	ns
Dead	51 (0.3%)	48 (94.1%)	3 (5.9%)	19 (39.6%)	3 (100.0%)	>0.05	ns	8 (16.7%)	0 (0.0%)	>0.05	ns
Unknown	2500 (17.1%)	2500 (100.0%)	0 (0.0%)	212 (8.5%)	N/A	N/A	N/A	0 (0.0%)	N/A	N/A	N/A

*Note:* The bold indicates that the analysis was for the whole group, separate from the individual analysis of sub-group categories relative to the indicated reference that are below the bold values.

Abbreviations: AHY, after hatch year; HY, hatch year; LBM, live bird market; ns, not significant.

^a^Categories marked “LBM” include only samples taken from live bird markets.

^b^The samples from Market 2 market were all quail.

*⁣* ^*∗*^, *p*  < 0.05;  ^*∗∗*^, *p*  < 0.01; *⁣*^*∗∗∗*^, *p*  < 0.001; *⁣*^*∗∗∗∗*^, *p*  < 0.0001.

**Table 5:** Double and triple stratification for age on variables for years 2017–2022.

Variable	Total samples^a^, no. (%)			Influenza A positive, no. (% positive per age)				H5 positive, no. (% positive per age)			
	*n*	HY	AHY	HY	AHY	*p*-value	Significance	HY	AHY	*p*-value	Significance
**Habitat**	**14,623**	—	—	—	—	—	—	—	—	—	—
Farm	689 (4.7%)	476 (69.1%)	213 (30.9%)	192 (40.3%)	100 (46.9%)	>0.05	ns	46 (9.7%)	0 (0.0%)	<0.0001	*⁣* ^*∗∗∗∗*^
Free-range farm	6009 (41.1%)	3007 (50.0%)	3002 (50.0%)	1072 (35.7%)	1171 (39.0%)	<0.01	*⁣* ^*∗∗*^	121 (4.0%)	65 (2.2%)	<0.0001	*⁣* ^*∗∗∗∗*^
LBM	5425 (37.1%)	4983 (91.9%)	442 (8.1%)	2308 (46.3%)	260 (58.8%)	<0.0001	*⁣* ^*∗∗∗∗*^	608 (12.2%)	135 (30.5%)	<0.0001	*⁣* ^*∗∗∗∗*^
Unknown	2500 (17.1%)	2,500 (100.0%)	0 (0.0%)	212 (8.5%)	N/A	N/A	N/A	0 (0.0%)	N/A	N/A	N/A
**Source of bird (LBM)^a^**	**7522**	—	—	—	—	—	—	—	—	—	—
Backyard farm	2677 (19.7%)	2080 (77.7%)	597 (22.3%)	958 (46.1%)	318 (53.3%)	<0.01	*⁣* ^*∗∗*^	409 (19.7%)	140 (23.5%)	<0.05	*⁣* ^*∗*^
Commercial farm	4725 (34.7%)	4635 (98.1%)	90 (1.9%)	1897 (40.9%)	57 (63.3%)	<0.0001	*⁣* ^*∗∗∗∗*^	364 (7.9%)	44 (48.9%)	<0.0001	*⁣* ^*∗∗∗∗*^
Hatchery	120 (0.9%)	120 (100.0%)	0 (0.0%)	16 (13.3%)	N/A	N/A	N/A	0 (0.0%)	N/A	N/A	N/A
**Sex**	**14,623**	—	—	—	—	—	—	—	—	—	—
Female	7101 (48.6%)	3904 (55.0%)	3197 (45.0%)	1504 (38.5%)	1254 (39.2%)	>0.05	ns	188 (4.8%)	70 (2.2%)	<0.0001	*⁣* ^*∗∗∗∗*^
Male	4147 (28.4%)	3687 (88.9%)	460 (11.1%)	1650 (44.8%)	277 (60.2%)	<0.0001	*⁣* ^*∗∗∗∗*^	555 (15.1%)	130 (28.3%)	<0.0001	*⁣* ^*∗∗∗∗*^
Unknown	3375 (23.1%)	3375 (100.0%)	0 (0.0%)	630 (18.7%)	0 (0.0%)	N/A	N/A	32 (0.9%)	0 (0.0%)	N/A	N/A
**Species**	**14,623**	—	—	—	—	—	—	—	—	—	—
Duck	7065 (48.3%)	3570 (50.5%)	3495 (49.5%)	1498 (42.0%)	1432 (41.0%)	>0.05	ns	417 (11.7%)	186 (5.3%)	<0.0001	*⁣* ^*∗∗∗∗*^
Chicken	4058 (27.8%)	3896 (96.0%)	162 (4.0%)	1635 (42.0%)	99 (61.1%)	<0.0001	*⁣* ^*∗∗∗∗*^	326 (8.4%)	14 (8.6%)	>0.05	ns
Quail	1000 (6.8%)	1000 (100.0%)	0 (0.0%)	436 (43.6%)	N/A	N/A	N/A	32 (3.2%)	N/A	N/A	N/A
Environmental (feces)	2500 (17.1%)	2500 (100.0%)	0 (0.0%)	212 (8.5%)	N/A	N/A	N/A	0 (0.0%)	N/A	N/A	N/A

*Note:* The bold indicates that the analysis was for the whole group, separate from the individual analysis of sub-group categories relative to the indicated reference that are below the bold values.

Abbreviations: AHY, after hatch year; HY, hatch year; LBM, live bird market; ns, not significant.

^a^Categories marked “LBM” include only samples taken from live bird markets.

*⁣* ^*∗*^, *p*  < 0.05; *⁣*^*∗∗*^, *p*  < 0.01;  ^*∗∗∗*^, *p*  < 0.001; *⁣*^*∗∗∗∗*^, *p*  < 0.0001.

## Data Availability

The data that support the findings of this study are openly available in Open Science Framework at https://osf.io/ehrpv/.
